# Transcriptome study of *Talaromyces verruculosus* TS63-9 for pectin-degrading CAZymes

**DOI:** 10.1128/spectrum.04069-25

**Published:** 2026-07-13

**Authors:** Liwei Hu, Yue Yang, Jerry Elorm Akresi, Yifan Zhang, Taibo Liang, Chenlin Miao, Qiang Xu, Xinpeng Zhang, Mengmeng Yang, Zongyu Hu, Yanbin Yin

**Affiliations:** 1 Zhengzhou Tobacco Research Institute of CNTC 154489, Zhengzhou, Henan province, China; 2 China Tobacco Jiangsu Industrial Co., Ltd 674981, Nanjing, Jiangsu province, China; 3 Department of Food Science and Technology, University of Nebraska-Lincoln 14719, Lincoln, Nebraska, USA; USDA-ARS San Joaquin Valley Agricultural Sciences Center, Parlier, California, USA

**Keywords:** pectin, *Talaromyces verruculosus*, CAZymes, endo-polygalacturonase

## Abstract

Pectins are a primary structural component of plant cell walls. In a previous study, we isolated a fungal strain, *Talaromyces verruculosus* TS63-9, which exhibited a high pectinase activity (142.43 U/mL) in a large-scale screening and sequenced its genome (GCA_001305275.1). Here, we further sequenced its transcriptome to study the transcriptional changes of TS63-9 in response to pectin treatment. A total of 1,435 genes were differentially expressed, with 757 upregulated and 678 downregulated. Among the differentially expressed genes, 47 were identified as encoding carbohydrate-active enzymes (CAZymes) for pectin degradation, including 39 upregulated and 8 downregulated genes. Notably, 18 upregulated CAZyme genes (including nine genes from GH28, and the rest from AA7, CE8, CE12, GH35, GH43, GH54, GH78, and PL1 families) contained a signal peptide, suggesting their potential secretion for extracellular pectin degradation. TS63-9 also possesses intracellular pectin catabolic genes (IPCGs) to encode enzymes for the further breakdown of pectin-derived monosaccharides, with 37 genes upregulated by pectins. Key pathways for D-galacturonic acid, L-rhamnose, and D-galactonate are strongly activated, alongside glycolysis and TCA cycle genes. This transcriptional activation demonstrates efficient carbon flux from pectin-derived monosaccharides into the central metabolism. Additionally, the promoters of 15 pectin-degrading CAZyme genes and 11 IPCGs contained a conserved pectin-responsive motif. Furthermore, the heterologous expression of a putative endo-polygalacturonase (endo-PG) gene in *Pichia pastoris* verified its function for pectin degradation. Our study provides the first insight into the transcriptional basis for the high pectinase activity of *T. verruculosus*.

**IMPORTANCE:**

Fungi are primary degraders of plant biomass in various environments by encoding and secreting numerous CAZymes. We previously isolated the fungus *Talaromyces verruculosus* TS63-9 from tobacco leaves with a high pectinolytic activity and sequenced its genome. In this study, we further sequenced its transcriptomes to identify and characterize pectin-degrading CAZymes. The extracellular pectinolytic CAZymes and the putative pectin-responsive motif identified from TS63-9 have great potential to be harnessed for sustainable bioenergy production and for enhancing various aspects of the agricultural industry.

## INTRODUCTION

Pectins, such as the water-soluble galacturonoglycans, are one of the major polysaccharide classes in plant primary cell walls (1). Many microorganisms, including bacteria and fungi, produce pectolytic enzymes (2–5), which have diverse industrial applications in textiles, paper processing, fruit juice extraction, and coffee and tea fermentation (6, 7). Based on their mode of action and substrate specificity, pectinases can be classified into three major groups: protopectinases, pectinesterases, and pectin depolymerases (8, 9). Among pectin depolymerases, pectate lyase (Pel, EC 4.2.2.2) has garnered significant scientific and commercial interest in recent decades. Pel cleaves α-1,4 linkages in pectate polymers via a β-elimination mechanism, generating 4,5-unsaturated oligogalacturonates (10). Industrial applications of bacterial alkaline Pels, predominantly from *Bacillus* and *Pseudomonas* bacteria, have been extensively studied (6, 11). In alkaliphilic *Paenibacillus* bacteria, several Pels also exhibit high stability (12, 13).

In fungi, *Penicillium* can also produce pectolytic enzymes. Recently, Liu et al. (14) sequenced the draft genome and secretome of *Penicillium decumbens*, a fungus used in industrial lignocellulolytic enzyme production (14). Their study revealed that compared to the widely used cellulase producer *Trichoderma reesei*, *P. decumbens* possesses a greater abundance of genes encoding lignocellulolytic enzymes—particularly those with carbohydrate-binding modules and hemicellulases. In addition to *P. decumbens*, *Penicillium verruculosum* is also a prominent lignocellulolytic fungus. This fungus produces multiple lignocellulolytic enzymes, including xyloglucanases (XG25 [25 kDa, pI 4.1, GH12] and XG70 [70 kDa, pI 3.5, GH74]) and cellulases (Cel5A [EG IIa, 39 kDa], Cel5B [EG IIb, 33 kDa], Cel6A [CBH II, 50–60 kDa], Cel7A [CBH I, 55–66 kDa], and Cel7B [EG I, 52–70 kDa]) (15). Notably, *P. verruculosum* WA30 demonstrated the highest cotton-degrading activity among 110 fungal species in a large-scale comparison (16). Another strain, *P. verruculosum* 28K, exhibited strong coregulated and enhanced production of carboxymethylcellulases, xylanases, β-glucanases, avicelases, and β-glucosidases, which are induced by celluloses and hemicelluloses (16).

In our previous work, we isolated the *T. verruculosus* strain TS63-9, which has the highest pectinase activity (142.43 U/mL) in a large-scale screening (17). Treatment of pipe tobacco with *T. verruculosus* fermentation broth reduced pectin content by up to 40%. We also sequenced the genome of *T. verruculosus* TS63-9 and identified all the carbohydrate-active enzymes (CAZymes) encoded in the genome (17). In this study, we employed Illumina sequencing to investigate the pectolytic CAZyme gene expression in *T. verruculosus* TS63-9 in response to pectin treatment. Additionally, we heterologously expressed a responsive polygalacturonase in *Pichia pastoris*, confirming its activity in pectin degradation.

## MATERIALS AND METHODS

### Strain characterization and culture media

*T. verruculosus* TS63-9 was isolated from flue-cured tobacco leaves and deposited and available in the China General Microbiological Culture Collection Center (CGMCC No. 6747, 2012.10.31). The strain was maintained on cornmeal medium as previously described (17).

To phylogenetically characterize *T. verruculosus* TS63-9, its genome was combined with other *Talaromyces* genomes available in GenBank and with genomes of *Penicillium chrysogenum* and *Aspergillus nidulans* for a whole-genome alignment using CLC Genomics Workbench (QIAGEN, version 23.0.4). A maximum likelihood phylogenetic tree was then constructed based on the aligned genome sequences using the Tree Construction tool with the Generalized Time-Reversible nucleotide substitution model and 1,000 bootstrap replicates in CLC Genomics Workbench. *P. chrysogenum* and *A. nidulans* were used as outgroups to root the tree.

### RNA sequencing and data analysis

Actively growing mycelia of TS63-9 were obtained by inoculating mycelial plugs (5 mm in diameter, taken from the leading edge of a 3-day-old potato dextrose agar [PDA] culture) into basic medium (Peptone 1.0 g/L, KH_2_PO_4_ 2.0 g/L, (NH_4_)_2_SO_4_ 1.4 g/L, Urea 0.3 g/L, MgSO_4_·7H_2_O 0.3 g/L, CaCl_2_ 0.3 g/L, FeSO_4_·7H_2_O 0.005 g/L, MnSO_4_·H_2_O 0.00156 g/L, ZnSO_4_·7H_2_O 0.0014 g/L, CoCl_2_·6H_2_O 0.002 g/L, and Tween-80 1 mL/L, pH 6.0). The pectin treatment group contains 5 g/L pectin (SKU: P9135-100G; Sigma-Aldrich, USA) added to the basic medium, while the control group received no pectin. After 24 h of incubation, the culture was centrifuged at 8,000 rpm for 5 min, and the pellet was collected in a 50 mL centrifuge tube. Each treatment group had three replicates.

Total RNA extraction was performed using the QIAGEN RNeasy Plant Mini Kit (QIAGEN, Germany). Integrity of RNA samples was verified using the 2100 Bioanalyzer (Agilent Technologies, Inc., Santa Clara, USA). To make RNA sequencing libraries, 1 μg total RNA per sample was used as input. Libraries were prepared with VAHTSTM mRNA-seq V2 Kit per the manufacturer’s guidelines. mRNA was purified using poly-T beads, fragmented, and reverse-transcribed into first-strand cDNA with random hexamers. Second-strand synthesis was followed by end repair, adenylation, and adaptor ligation. Index codes were added for sample multiplexing. A paired-end library (500 bp insert size) was constructed, and sequencing was performed on an Illumina HiSeq 2500 using a PE150 strategy (Sangon Biotech, Shanghai, China). Raw reads underwent quality control, including adapter trimming and low-quality read removal. Filtered reads were mapped to the genome of *T. verruculosus* TS63-9 using CLC Genomics Workbench (QIAGEN, Aarhus, Denmark) to study the expression of 11,326 annotated protein-coding genes. Gene expression levels were quantified as transcripts per million (TPM). Differentially expressed genes (DEGs) were identified using thresholds of FDR-adjusted *P* value <0.05 and |log₂FC| > 1 (2-fold change). Functional annotation and enrichment analysis were conducted via the eggNOG-mapper web server (18).

### Cell wall-degrading CAZyme and pectinase analysis

The predicted protein sequences were analyzed for CAZymes using the CAZy database (19) and the dbCAN3 web server (20). CAZyme domain identification was based on the following three HMMER search parameters: E-value, alignment length, and sequence coverage. Stringent thresholds were applied, requiring an E-value of <1 × 10⁻⁵ for alignments longer than 80 amino acids and <1 × 10⁻³ for shorter alignments (≤80 amino acids). Glycosyltransferase (GT) domains were excluded from subsequent analyses, as they are not for carbohydrate degradation.

### Intracellular pectin catabolic genes analysis

The intracellular pectin catabolic genes (IPCGs) in *Aspergillus niger* ATCC 1015/NRRL3 were reported previously (21). To identify IPCGs in TS63-9, OrthoFinder v 2.5.5 (22) was run to identify orthologs in the genomes of TS63-9 (GCA_001305275.1) and ATCC 1015/NRRL3 (GCA_000230395.2). By parsing the OrthoFinder result, the corresponding genes in TS63-9 that are orthologous to IPCGs of NRRL3 genes were extracted.

The TS63-9 genome was downloaded from NCBI (GCA_001305275.1), including the genome sequence file (fna), the protein sequence file (faa), and the gene position file (gff). DEGs that are CAZymes and IPCGs were examined for their genomic positions. From the transcription start site of each gene (from the gff file), the 2,000 bp upstream regions were extracted as the promoter regions. Putative motifs in these promoter regions were identified using MEME Suite Programs (23).

### Detailed analysis of CAZyme family GH28

The GH28 protein domains were analyzed using both dbCAN3 and Swiss-Prot databases (24). Signal peptide prediction was performed using the SignalP 6.0 online server (25). Domain architecture visualization was generated with DOG 2.0 (26), while three-dimensional protein structure modeling was conducted using the AlphaFold server (27). Phylogenetic analysis of GH28 protein sequences was performed using the maximum likelihood algorithm with the Whelan and Goldman model for amino acid substitutions with 100 bootstrap replicates (28).

### Heterologous expression of endo-polygalacturonase in *Pichia pastoris*

The endo-polygalacturonase (PG) gene of TS63-9 without the signal peptide (1–19 aa), labeled with a His-tag (HHHHHH), was commercially synthesized by Sangon Biotech (Shanghai) Co., Ltd. The synthetic gene was then digested with NotI and EcoRI, and the resulting fragment was ligated into the pPICZαA vector and transformed into *Escherichia coli*-competent cells. Positive clones were selected for sequencing validation. Verified positive clones were inoculated into LB liquid medium and incubated overnight with shaking for plasmid extraction. Positive clones were cultured in LB liquid medium with overnight shaking. Plasmid DNA was extracted and transformed into competent *P. pastoris* X33 yeast cells following the manufacturer’s protocol (Invitrogen). Successful insertion of the PG gene was verified by PCR using primer set AOX5′-F/AOX3′-R. The PCR reaction mixture (20 μL total volume) contained 2 × Fast Taq mix (7.5 μL), 10 μM AOX5′ primer (1 μL), 10 μM AOX3′ primer (1 μL), Template DNA (1 μL), and ddH₂O (9.5 μL). Amplification conditions were initial denaturation at 95°C for 5 min; 30 cycles of 94°C for 30 s, 52°C for 30 s, and 72°C for 90 s, followed by final extension at 72°C for 5 min. PCR products were analyzed by electrophoresis on a 1.5% agarose gel at 150 V for 15 min. Transformed yeast clones were screened for pectinase activity as described previously (29–31) with minor modifications. Specifically, transformed yeast clones were screened for pectinase activity on MP-5 plates containing 0.5% pectin and 1% methanol. Methanol was supplemented every 24 h. The plates were then inoculated with the transformed yeast clones and incubated at 30°C for 3 days. After 3 days of cultivation, 1% CTAB solution was gently poured onto the plate surface to observe the formation of a clear halo, indicating pectinase activity. A yeast strain transformed with an empty pPICZαA vector was used as the negative control.

## RESULTS

### Morphological and phylogenetic characterization of *T. verruculosus* TS63-9

**Fig 1:**
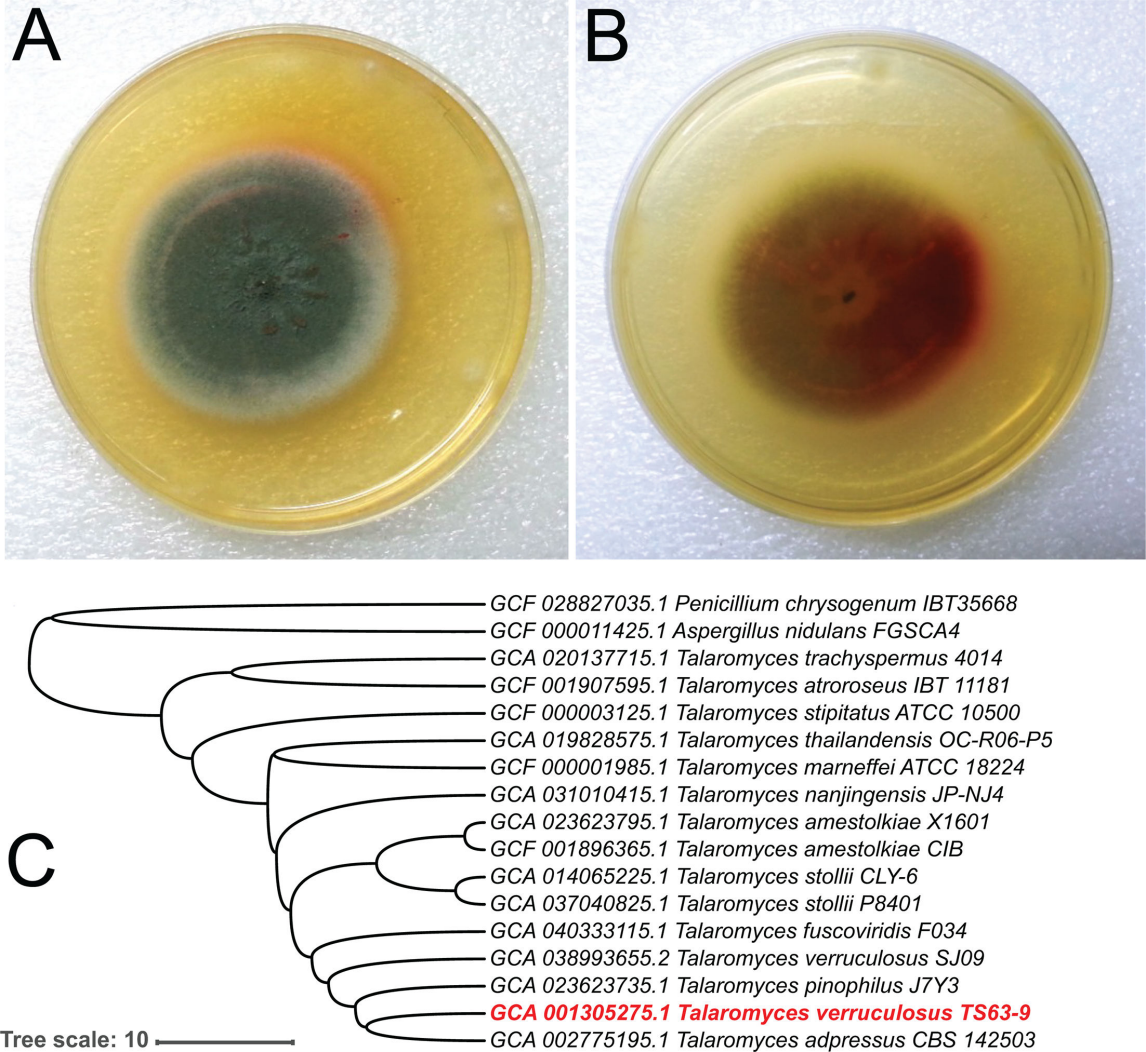
Characterization of *T. verruculosus* TS63-9. (**A**) Upward view of *T. verruculosus* TS63-9 growing on a PDA plate. (**B**) Downward view. (**C**) Maximum likelihood phylogeny of 15 *Talaromyces* genomes and two outgroups (*P. chrysogenum* and *A. nidulans*) using their whole-genome alignment. All nodes have bootstrap values >80%.

*T. verruculosus* TS63-9 was grown on PDA plates. The colonies exhibited a circular morphology with cyan-colored, villous centers and white margins (Fig. 1A). Notably, the strain secreted a red pigment that diffused into the medium, resulting in a dark red coloration (Fig. 1B).

We previously sequenced the 18S rRNA sequence (KT897711.1) of TS63-9 (17). Using BLASTN, we gathered closely related 18S rRNA sequences in GenBank and built an 18S rRNA phylogeny (Fig. S1). *T. verruculosus* TS63-9 clusters closely with *T. flavus* var. flavus (NG_062802.1), *T. viridis* (NG_062609.1), and *T. aerugineus* (NG_062662.1). In addition to the genome of *T. verruculosus* TS63-9, additional *Talaromyces* genomes are also available in GenBank. We further built a whole genome alignment for 15 *Talaromyces* genomes and two outgroup genomes and built a genome phylogeny (Fig. 1C), which further demonstrated its closest genome-level similarity to *Talaromyces adpressus* CBS 142503 (32).

### Transcriptomic response of TS63-9 to pectin treatment

**Fig 2:**
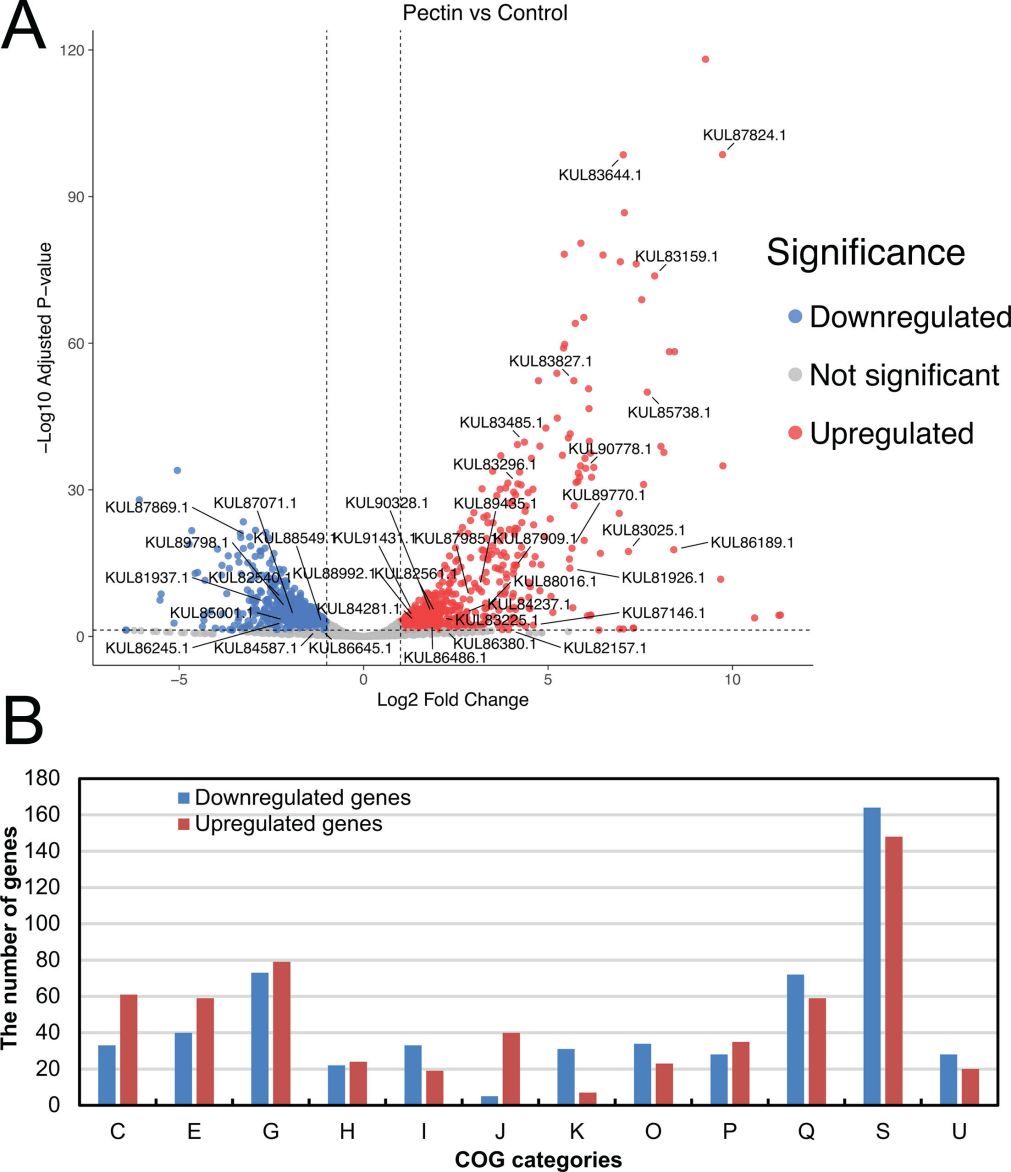
Differential gene expression of *T. verruculosus* TS63-9 under pectin treatment. (**A**) Volcano plot of differential gene expression. Related differentially expressed pectin-degrading CAZymes are labeled. The complete list of the 1,435 differentially expressed genes can be found in Table S2. (**B**) Top COG (Database of Clusters of Orthologous Genes) functional categories sorted by the number of upregulated genes. Only functional categories with >20 upregulated genes are shown. The COG functional categories are as follows: C, energy production and conversion; E, amino acid transport and metabolism; G, carbohydrate transport and metabolism; H, coenzyme transport and metabolism; I, lipid transport and metabolism; J, translation, ribosomal structure, and biogenesis; K, transcription; O, post-translational modification, protein turnover, chaperones; P, inorganic ion transport and metabolism; Q, secondary metabolites biosynthesis, transport and catabolism; S, function unknown; and U, intracellular trafficking, secretion, and vesicular transport.

A total of 79.64 GB of clean RNA-seq data were generated for three control and three pectin treatment samples (average 13.27 GB per sample; Table 1). Mapping the clean reads to the TS63-9 genome (GCA_001305275.1) found that between 95.57% and 96.51% of reads were mapped in the six samples. Out of the 11,326 protein-coding genes encoded in the genome, 11,028 genes were expressed in at least one condition (Table S1). Using stringent thresholds (fold-change ≥2 in TPM values, FDR-adjusted *P* value < 0.05), we identified 1,435 DEGs (Fig. 2A), including 678 upregulated and 757 downregulated genes following the pectin treatment (Table S2).

**TABLE 1:** RNA-seq reads in six samples of *P. verruculosum* TS63-9

Samples	Data size (GB)	Clean read count	Mapped read count	Mapped read, %
C1	12.56	37,456,716	36,047,268	96.24%
C2	14.10	42,000,244	40,535,423	96.51%
C3	13.74	40,963,158	39,369,169	96.11%
T1	13.54	40,375,658	38,656,766	95.74%
T2	12.98	38,678,054	37,007,672	95.68%
T3	12.72	37,887,828	36,208,455	95.57%

The top functional categories with the most DEGs (Fig. 2B) were (G) carbohydrate transport and metabolism, (C) energy production/conversion, (E) amino acid transport and metabolism, and (Q) secondary metabolites biosynthesis/transport/catabolism. While there were more downregulated (757) than upregulated (678) genes, the following functional categories contain more upregulated genes: (G) carbohydrate transport and metabolism, (C) energy production/conversion, (E) amino acid transport and metabolism, (J) translation/ribosomal structure/biogenesis, and *(*P) inorganic ion transport and metabolism. In contrast, more downregulated genes were found in the (Q) secondary metabolites biosynthesis/transport/catabolism, (I) lipid transport and metabolism, and (O) post-translational modification, protein turnover, and chaperone categories. This observation is expected and suggests that the pectin treatment activated the expression of more genes to utilize pectins for fungal growth and development.

### Pectin-degrading CAZymes and intracellular pectin catabolic enzymes with differential gene expression

Among the 1,435 DEGs, we identified 47 genes encoding pectin-degrading CAZymes (Table S3), which include two carbohydrate esterase families (CE8 [three genes] and CE12) (1), 12 glycoside hydrolase families (GH28 (11), GH2 (5), GH43 (4), GH78 (4), GH35 (3), GH31 (2), GH51 (2), GH54 (2), GH127 (2), GH105 (1), GH53 (1), GH139 (1)), one pectate lyase family (PL1 (1)), and one auxiliary activity family (AA7 (5)). These CAZyme families were determined as pectin-degrading according to a recent review paper and dbCAN/CAZy annotation (19, 20, 33). These include important enzymes for pectin backbone and side chain degradation, such as GH28 for polygalacturonases and rhamnogalacturonases, GH35 for galactanases, GH43 for arabinanases, GH78 for rhamnohydrolases, GH31 for xylosidases, GH51 and GH54 for arabinofuranosidases, GH127 for arabinobiosidases, GH139 for fucosidases, and AA7 for galacturonide oxidases. Altogether, these 47 CAZyme genes include 39 upregulated and 8 downregulated genes under pectin treatment (Table 2). Notably, 23 CAZymes (18 upregulated) possess signal peptides, suggesting extracellular secretion for external pectin degradation.

**TABLE 2:** Forty-seven differentially expressed pectin-degrading CAZymes in TS63-9^*c*^

Gene ID	Protein ID	Fold change^*a*^	Adj *P* value	dbCAN domains^*b*^	Predicted EC	Signal peptide
ZTR_03139	KUL87824.1	850.01	2.60E-99	**GH28_e58**	3.2.1.67|3.2.1.-	Y
ZTR_08398	KUL86189.1	339.5	1.79E-18	**GH28_e27**	3.2.1.171	N
ZTR_00196	KUL90781.1	267.73	1.30E-39	GH78_e52	3.2.1.174	Y
ZTR_10118	KUL83159.1	237.92	1.70E-74	**GH28_e75**	3.2.1.15	Y
ZTR_08527	KUL85738.1	206.95	1.03E-50	**CE8_e16 + GH28_e56**	3.2.1.15|3.1.1.11	Y
ZTR_09302	KUL83025.1	145.17	4.25E-18	CE8_e105 + CE8_e105	3.1.1.11	Y
ZTR_11056	KUL83644.1	131.99	2.80E-99	GH35_e22 + CBM67_e19	–	Y
ZTR_10807	KUL83492.1	89.74	9.17E-79	GH2_e61	3.2.1.23	N
ZTR_00193	KUL90778.1	64.88	3.91E-35	**GH28_e68**	–	Y
ZTR_06634	KUL83827.1	51.88	4.77E-53	GH43_e271 + CBM91_e16	–	N
ZTR_00500	KUL89770.1	50.26	9.08E-19	PL1_e51	4.2.2.2	Y
ZTR_10728	KUL81926.1	48.06	1.13E-14	**GH28_e56**	3.2.1.15|3.1.1.11	Y
ZTR_04293	KUL89416.1	24.34	1.47E-23	CBM67_e28 + GH78_e80	3.2.1.40|3.1.1.73	N
ZTR_05615	KUL87146.1	24.24	0.00,495	GH43_e312	–	Y
ZTR_00059	KUL90550.1	21.24	1.96E-27	GH105_e57	–	N
ZTR_09329	KUL83001.1	19.51	5.12E-24	AA7_e0	1.1.3.-	N
ZTR_11349	KUL83296.1	18.07	5.95E-32	**GH28_e75**	3.2.1.15	N
ZTR_11088	KUL82157.1	15.25	0.04	**GH28_e56**	3.2.1.15|3.1.1.11	Y
ZTR_01887	KUL90342.1	13.1	2.16E-09	GH139_e1	–	N
ZTR_03993	KUL88016.1	9.8	1.97E-06	**GH28_e75**	3.2.1.15	Y
ZTR_04324	KUL89435.1	8.74	2.04E-10	**GH28_e93**	3.2.1.-	Y
ZTR_03944	KUL87985.1	7.38	3.02E-08	GH35_e18 + CBM67_e19	3.2.1.-	N
ZTR_06924	KUL84237.1	6.32	2.62E-05	GH53_e5	3.2.1.89	N
ZTR_07060	KUL83956.1	4.99	1.49E-11	AA7_e0	1.1.3.-	Y
ZTR_08139	KUL86380.1	4.52	0.02	GH51_e35	3.2.1.55	N
ZTR_04255	KUL86505.1	4.15	3.64E-11	GH2_e138	3.2.1.25	N
ZTR_10471	KUL83225.1	4.1	0.000,108	CE12_e43	3.1.1.-	Y
ZTR_09370	KUL82561.1	3.94	4.98E-05	**GH28_e79**	–	Y
ZTR_02074	KUL90328.1	3.66	6.67E-05	GH35_e4 + CBM67_e19	3.2.1.23|2.4.1.-	N
ZTR_10587	KUL83549.1	3.09	0.00,142	CBM67_e14 + GH78_e23	–	N
ZTR_03261	KUL87777.1	3.05	0.000,006	GH2_e138	3.2.1.25	N
ZTR_10403	KUL82929.1	2.78	3.37E-04	AA7_e0	1.1.3.-	N
ZTR_07561	KUL84281.1	2.72	0.00,238	GH54_e0 + CBM42_e0	3.2.1.55|3.2.1.37	Y
ZTR_06089	KUL88992.1	2.67	0.000,354	CE8_e26	–	Y
ZTR_10463	KUL83249.1	2.66	0.00,939	CBM67_e28 + GH78_e80	3.2.1.40|3.1.1.73	N
ZTR_01529	KUL91431.1	2.54	0.000,018	GH51_e31	3.2.1.55	N
ZTR_10417	KUL82901.1	2.35	0.03	GH2_e138	3.2.1.25	N
ZTR_01775	KUL91441.1	2.1	0.04	GH127_e10	–	N
ZTR_04499	KUL89458.1	2.01	2.66E-03	AA7_e0	1.1.3.-	N
ZTR_00954	KUL91081.1	−2.23	0.00,607	GH127_e0	3.2.1.185	N
ZTR_08648	KUL83731.1	−2.33	8.75E-05	GH31_e7	3.2.1.20|3.2.1.84	Y
ZTR_03705	KUL89160.1	−2.38	0.00,542	GH31_e7	3.2.1.20|3.2.1.84	N
ZTR_01456	KUL91907.1	−2.82	5.73E-05	GH2_e60	3.2.1.165	Y
ZTR_00466	KUL89798.1	−4.15	4.53E-06	GH43_e158	–	N
ZTR_09181	KUL82540.1	−4.45	4.40E-08	GH54_e0 + CBM42_e0	3.2.1.55|3.2.1.37	Y
ZTR_10342	KUL81937.1	−5.94	1.31E-07	GH43_e126 + CBM91_e7	–	Y
ZTR_00569	KUL89755.1	−19.82	2.96E-05	AA7_e0	1.1.3.-	Y

^*a*^ Positive and negative numbers indicate the genes have (at least 2-fold) higher or lower expression in pectin treatment, respectively, compared to control treatment.

^*b*^ dbCAN-sub was used in the search for dbCAN subfamilies (Table S3).

^*c*^ "–" indicates no predicted EC number and GH28 family members are indicated in bold.

The extracellular breakdown of pectins by CAZymes produces various monosaccharides for further intracellular catabolism for energy production and fungal growth. The intracellular catabolism of pectin breakdown products contains multiple known metabolic pathways, for example, the pentose catabolic pathway (PCP), pentose phosphate pathway (PPP), other sugar-specific metabolic pathways (e.g., D-galactose, D-mannose, D-gluconate, D-galacturonic acid, L-rhamnose, and glycerol metabolism), as well as the central carbon catabolic pathways (e.g., glycolysis, tricarboxylic acid [TCA] and glyoxylate cycles). Enzymes in these pathways have been reported in *Aspergillus niger* and a few other model fungal organisms (21). By comparison with these IPCGs in *A. niger*, we identified their orthologs in the TS63-9 genome.

Among 148 IPCGs in TS63-9, 30 genes were upregulated, and 7 were downregulated under pectin treatment (Table S4). For example, all genes in the D-galacturonic acid pathway were strongly activated, including *gaaA* (D-galacturonic acid reductase), *gaaC* (2-keto-3-deoxy-L-galactonate aldolase), *gaaB* (gluconate/galactonate dehydratase), and *garB* (D-galacturonate reductase). Three genes in the L-rhamnose pathway were upregulated, including *lraA* (L-rhamnose-1-dehydrogenase), *lrdA* (L-rhamnonate dehydratase), and *lkaA* (L-2-keto-3-deoxyrhamnonate aldolase). Additionally, transcriptional upregulation of multiple genes in the glycolysis (e.g., fbaA: fructose-bisphosphate aldolase) and TCA cycle pathways (e.g., pdaA: pyruvate dehydrogenase complex, alpha subunits) suggests efficient carbon flux from sugar monomers into the central carbon catabolic metabolism (Table S4). Overall, TS63-9 is equipped with all major pathways and transcriptionally activated to efficiently utilize pectin breakdown products intracellularly.

### Functional domain analysis of GH28 CAZymes

**Fig 3:**
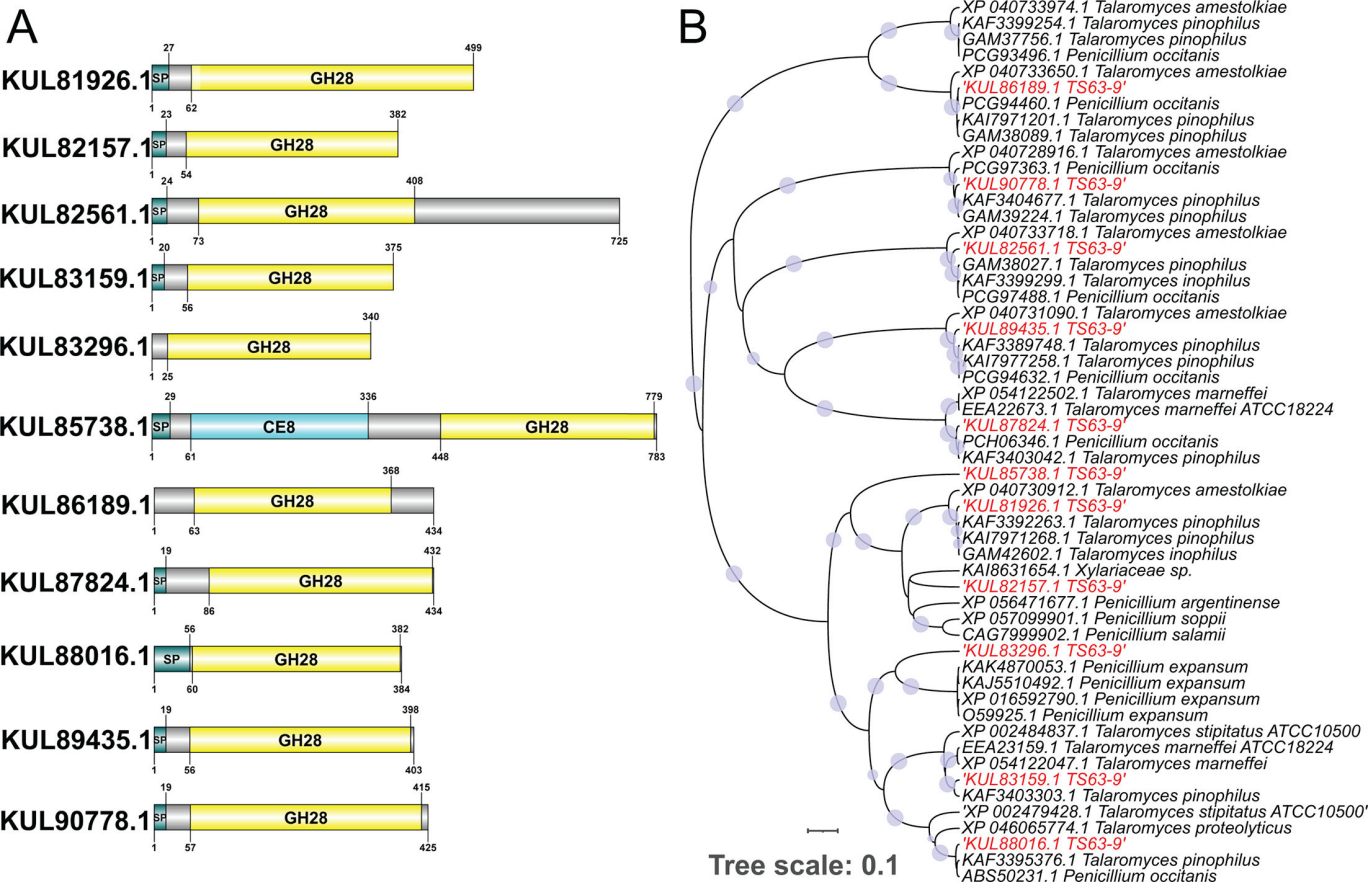
Eleven upregulated GH28 proteins in TS63-9. (**A**) CAZyme domain architectures with signal peptides. (**B**) Maximum likelihood phylogeny of GH28 proteins in TS63-9 and closely related *Talaromyces* genomes. All highlighted nodes have bootstrap values >80%.

A total of 11 GH28 family genes were upregulated by pectin treatment, and most of them had the highest fold changes (e.g., FC > 10) among all the differentially expressed pectin-degrading CAZymes (Table 2). Signal peptides were identified in nine of these GH28 proteins, except for KUL83286.1 and KUL86189.1 (Fig. 3A), suggesting their potential role in extracellular pectin degradation. Notably, KUL85738.1 contains two CAZyme domains: GH28 (EC: 3.2.1.15) and CE8 (EC: 3.1.1.11). According to the best matches of these GH28 proteins in the CAZy database (19), EC functions (Table 2) were also predicted for galacturonan endo-α-1,4-galacturonidases (3.2.1.15), galacturonan exo-α-1,4-galacturonidases (3.2.1.67), rhamnogalacturonan I endo-α-1,2-galacturonidases (3.2.1.171), and xylogalacturonan endo-α-1,4-galacturonidase (3.2.1.-).

To study how the 11 GH28 genes are evolutionarily related, we searched for their protein homologs in GenBank (E-value <0.001). A phylogeny using the Neighbor-Joining algorithm was built for GH28 proteins from both TS63-9 and other *Talaromyces* genomes (Fig. 3B). The TS63-9 GH28 proteins were distributed throughout the phylogenetic tree, and each was clustered with its close orthologs in other *Talaromyces* species. This distribution pattern suggests that different GH28 gene copies have evolved before the divergence of different *Talaromyces* species.

### Putative motif in promoters of differentially expressed pectin-degrading CAZyme genes

A potential pectin-responsive regulatory motif (CCACCA) was identified by bioinformatic analysis in the promoter regions of 15 out of the 47 differentially expressed CAZyme genes from TS63-9 (Table 3 and Fig. S2). These 15 CAZymes (14 upregulated) include key pectin-degrading enzymes such as GH28 for polygalacturonases and rhamnogalacturonases, GH35 and GH53 for galactanases, GH43 for arabinanases, GH78 for rhamnohydrolases, PL1 for pectin lyases, GH51 for arabinofuranosidases, GH139 for fucosidases, and CE12 for pectin acetylesterases. This motif was also found in 11 differentially expressed IPCGs, among which 10 were upregulated under pectin treatment. These included genes in various pathways of the intracellular pectin catabolism, such as the D-galacturonic acid pathway (*gaaABC*), the L-rhamnose pathway (*lraA*), the glycerol pathway, as well as PCP, np-ED (Non-phosphorylated DeLey-Doudoroff pathway), and TCA (tricarboxylic acid) pathways (Table 3).

**TABLE 3:** Conserved motif in promoters of pectin-degrading CAZymes and intracellular pectin catabolic genes

Gene ID	Protein ID	Fold change	dbCAN domains or IPCG pathways^*b*^^,*c*^	Strand	Position from start codon	Motif sequence^*a*^
ZTR_03139	KUL87824.1	850.01	**GH28**	−	−513	TT**CCCCCA**AT
					−544	TT**CCACCC**AT
ZTR_00196	KUL90781.1	267.73	GH78	−	−320	TT**CCACCA**AT
ZTR_11056	KUL83644.1	131.99	GH35 +CBM67	−	−224	TT**CCACCA**AT
					−392	AT**CCCCCA**AT
ZTR_06634	KUL83827.1	51.88	GH43 +CBM91	−	−130	TT**CCATCA**AT
					−153	AT**CCACCA**AT
ZTR_00500	KUL89770.1	50.26	PL1	+	−507	TT**CCACAA**AT
					−752	TT**CCACCA**GT
ZTR_11088	KUL82157.1	15.25	**GH28**	−	−147	TT**CCACCA**AT
					−404	AT**CCATCC**AT
					−420	AT**CCATCC**AT
ZTR_01887	KUL90342.1	13.1	GH139	−	−70	TC**CCAGCA**AT
ZTR_03993	KUL88016.1	9.8	**GH28**	−	−216	TG**CCACCA**AT
ZTR_04324	KUL89435.1	8.74	**GH28**	−	−157	TT**CCACCA**AA
					−367	AA**CCACCA**AT
ZTR_06924	KUL84237.1	6.32	GH53	−	−154	TT**CCAGCA**AA
ZTR_08139	KUL86380.1	4.52	GH51	−	−519	AT**CCAGCA**AA
ZTR_10471	KUL83225.1	4.1	CE12	+	−154	AC**CCACCC**AT
					−303	TT**CCCCCA**AT
ZTR_09370	KUL82561.1	3.94	**GH28**	−	−75	TT**CCACCC**AT
ZTR_03261	KUL87777.1	3.05	GH2	−	−123	TT**CCACCA**AT
ZTR_10342	KUL81937.1	−5.94	GH43 +CBM91	+	−51	AT**CCCCCA**AT
ZTR_05992	KUL89057.1	168.04	D-GalA (*gaaB*)	−	−435	TT**CCACCA**AT
ZTR_00179	KUL90469.1	124.25	D-GalA (*gaaC*)	+	−277	TT**CCACCA**AT
ZTR_00164	KUL90656.1	42.8	D-GalA (*gaaA*)	−	−598	TT**CCACCA**AT
ZTR_03366	KUL87216.1	10.27	L-Rha (*lraA*)	+	−77	AT**CC**C**CCA**AT
ZTR_04172	KUL86597.1	5.82	Glycerol (EC 1.1.5.3)	−	−608	TC**CCACCA**AT
ZTR_10891	KUL82777.1	4.37	PCP (*ladA*)	+	−859	AT**CC**G**CCA**AT
ZTR_07195	KUL84128.1	2.81	TCA (EC 1.2.1.-)	+	−106	TT**CCA**T**CA**AT
ZTR_01550	KUL91472.1	2.67	PCP (*lxrA*)	+	−347	AT**CCACCA**CA
ZTR_00702	KUL90887.1	2.41	np-ED (*dakA*)	+	−871	TT**CCA**T**CA**CT
ZTR_07383	KUL85832.1	2.32	TCA (*pdaA*)	+	−316	TT**CCACCA**CT
ZTR_03722	KUL89175.1	−6.81	D-gluconate (*gox*)	+	−277	TT**CCA**T**C**CAT

^*a*^ The core motif is shown in boldface with two flanking bases up- and down-stream. Within the core motif, bases matching the most occurred instance ***CCACCA*** are underlined*.*

^*b*^ dbCAN domains are shown for CAZymes, while pathways (gene names or ECs) are shown for IPCGs (intracellular pectin catabolic genes): D-GalA, D-galacturonic acid; L-Rha, L-Rhamnose; PCP, pentose catabolic pathway; np-ED, non-phosphorylated DeLey-Doudoroff pathway; and TCA , tricarboxylic acid.

^*c*^ GH28 family members are indicated in bold.

The motif has different variants, and their instances are located 51–871 bp upstream of the coding regions of the 15 CAZymes and 11 IPCGs, sometimes with multiple occurrences. For example, the GH28 gene ZTR_11088 contained three motif instances including “CCACCA,” “CCATCC,” and “CCATCC.” The most occurred motif variant, “CCACCA,” is found in the promoter regions of eight CAZyme genes and six IPCGs. Given that these findings are derived solely from *in silico* promoter analysis, future experimental validation (e.g., via electrophoretic mobility shift assays, promoter-reporter fusions, or gene deletion studies) is required to validate the regulatory function of this motif in coordinating the co-regulation of these pectin-degrading genes.

### Heterologous expression of an exo-α-1,4-galacturonidases ZTR_10118

Endo-polygalacturonases (endo-PGs) from the GH28 family cleave the α-1,4-linked galacturonic acid backbone in homogalacturonan (HG), while other GH28 members include exo-polygalacturonosidases (exo-PGs) and enzymes targeting the α-1,2-linked galacturonic acid-rhamnose backbone of rhamnogalacturonan I (RG-I). These enzymes play crucial roles in fungal pectin degradation. Notably, the endo-α-1,4-galacturonidase (endo-PG) gene ZTR_10118 (EC 3.2.1.15) showed a remarkable upregulation (FC = 237.92, Table 2) upon pectin treatment. Its protein product (KUL83159.1) is 375 aa long with an N-terminal signal peptide and a GH28 domain (56–375 aa) (Fig. 3A). It shares 76% sequence identity and similar sequence length with its best hit in the Swiss-Prot database, the Polygalacturonase 2 (PG2, UniProt ID: Q9Y833.1, length: 380 aa) of *Penicillium olsonii* (Table S5). The PG2 protein from *P. olsonii* has been cloned and purified, showing acidic PG activity strongly induced by pectins (34). Although its specific activity was not determined, PG2 is annotated with EC 3.2.1.15 (endo-PG) in the CAZy database. Therefore, we infer that KUL83159.1 also has endo-PG activity.

**Fig 4:**
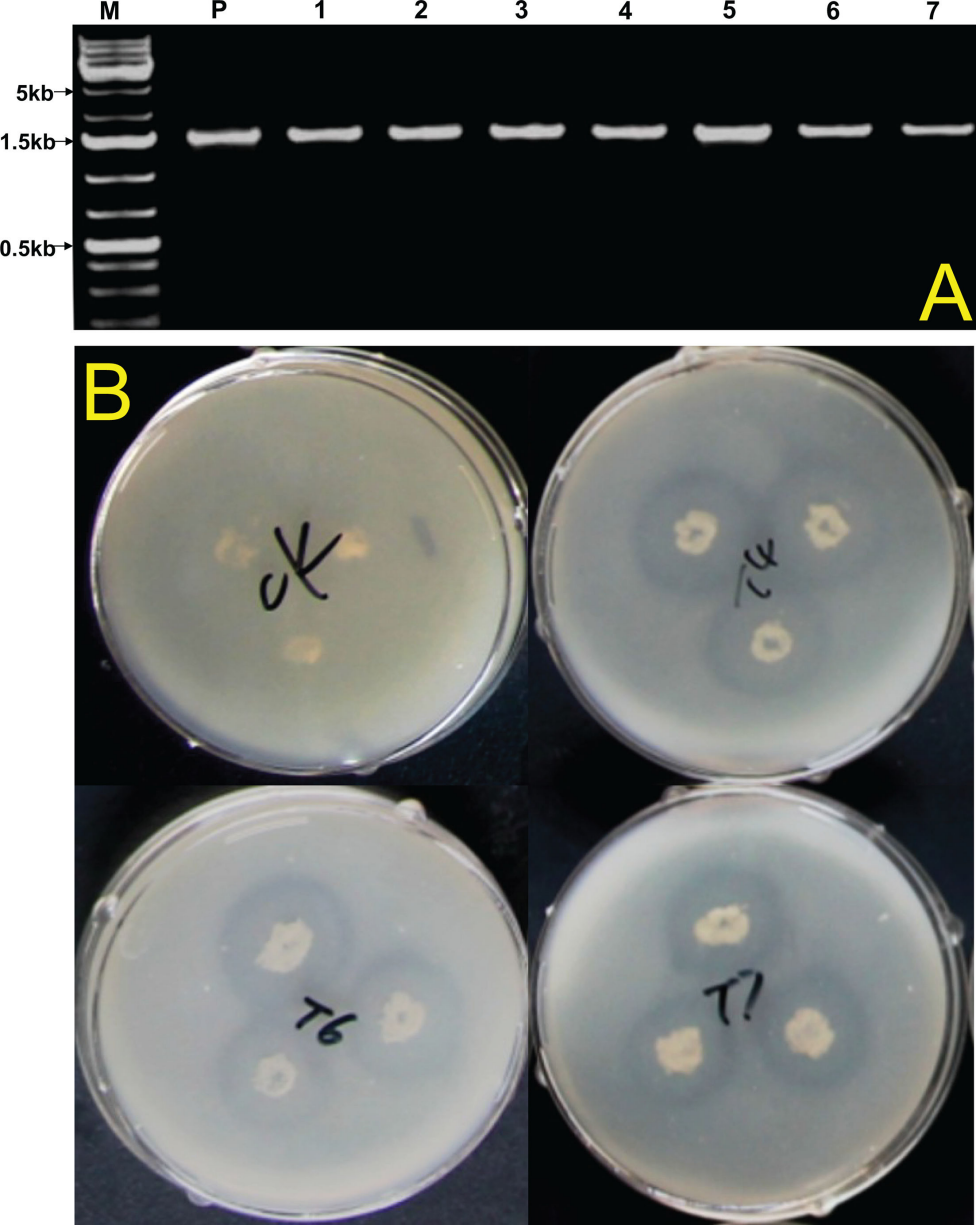
Experimental verification of the gene expression of the endo-PG gene (ZTR_10118). (**A**) PCR confirmation of transformed yeast (1,635 bp). Note: M, marker; P, positive control; 1–7, picked colonies. (**B**) Screening of yeast containing pectinase activity in plates with the MP-5 medium, which is a commonly used growth medium for detecting pectinolytic microorganisms, especially those producing polygalacturonase. CK, Control plate (pPICZαA vector without endo-PG gene transformed yeast) and T (4, 6, 7). Three plates each with three positive colonies (pPICZαA vector with endo-PG gene transformed yeasts).

To verify the expression of this endo-PG and its functional role in pectin degradation, we synthesized the mature coding sequence (without signal peptide) of KUL83159.1 and cloned it into the pPICZαA vector. Successful transformation of the vector into yeast (*P. pastoris*) was confirmed by a PCR amplification using AOX5′/AOX3′ primers (Fig. 4A). Growing the transformed yeasts on plates with pectins confirmed that the transformed yeasts grew well with clear cell lawns (hydrolysis zones), while the control plates (non-transformed yeasts) had no cell lawns (Fig. 4B). This experiment verified that the endo-PG enzyme of TS63-9 was indeed expressed, and its expression enabled the yeast growth on pectins. However, deep enzymatic characterization with the purified endo-PG (ZTR_10118) protein will be required in the future to confirm its pectinolytic activity.

## DISCUSSION

**Fig 5:**
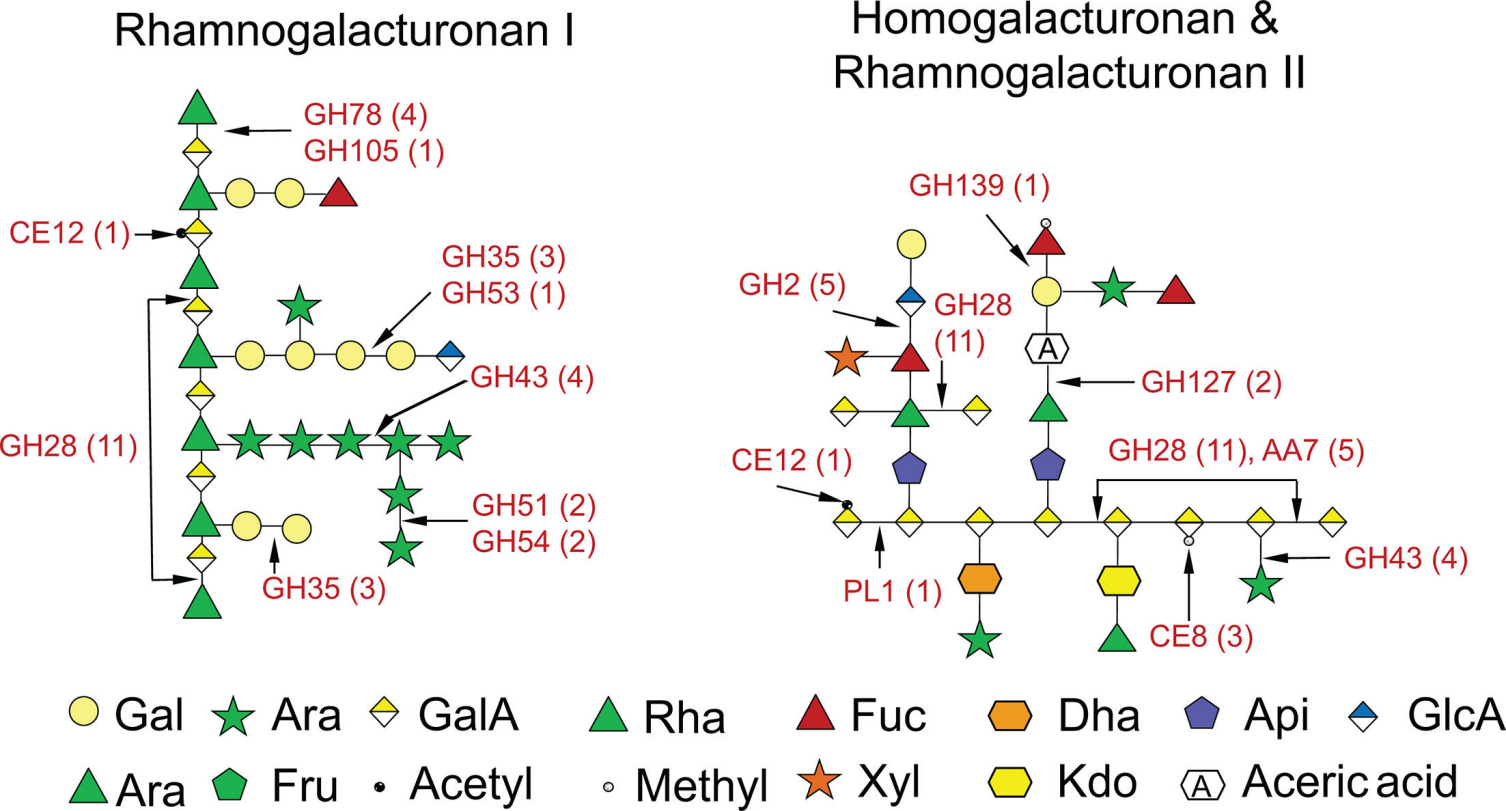
A model for pectin degradation in *T. verruculosu*s TS63-9. Diagram showing putative cleavage sites of Homogalacturonan and Rhamnogalacturonan I & II for differentially expressed pectin-degrading CAZymes (Table 2). This model is generated based on the synthesis of knowledge published in recent papers and curated from dbCAN/CAZy annotation (19, 20, 33, 46, 47).

Pectins represent a complex family of plant cell wall polysaccharides comprising several structural classes: homogalacturonan (HG), xylogalacturonan (XGA), apiogalacturonan (AGA), rhamnogalacturonan I (RG-I), and rhamnogalacturonan II (RG-II) (35–38). Among these, HG, RG-I, and RG-II constitute the most structurally complex components (36, 39). HG, accounting for >60% of pectin content, consists of α-1,4-linked D-galacturonic acid polymers (40), while RG-II, although representing only ~4%–8% of cell wall mass (35, 41, 42), has a HG backbone and displays a remarkable structural complexity in its side chains (Fig. 5). RG-I features an alternating α-1,2-rhamnosyl and α-1,4-galacturonic acid backbone decorated with diverse side chains, including α-1,5-arabinans and arabinogalactans (35, 38). Both HG and RG-I may undergo methyl esterification or acetylation (35, 38, 40). Overall, pectins are the most structurally complex carbohydrates in plant cell walls (43) and require numerous plant CAZymes for their synthesis (44). Given their structural complexities, the microbial degradation of different classes of pectins also demands a high diversity of pectinases such as hydrolases, depolymerases, esterases, and lyases enzymes (33). Pectinases have various industrial applications (45) such as converting agro-wastes into high-value bioproducts in the bioenergy industry, increasing the quality of wine, beer, tea, coffee, and juice in the food industry, as well as fiber retting in the textile industry. Fungi are sources of many highly efficient and commercially exploited industrial CAZymes such as cellulases, amylases, laccases, xylanases, and pectinases. This study has a goal to search for differentially expressed pectinases in *T. verruculosus* TS63-9, which was shown to have high pectinase activity (142.43 U/mL) in a large-scale screening (17).

Our RNA-seq data analysis revealed that pectin treatment significantly changed the expression of 1,435 genes in *T. verruculosus* TS63-9 and predominantly activated the carbohydrate/amino acid transport/metabolism and energy production pathways (Fig. 2). Notably, 39 genes encoding 15 pectin-degrading CAZyme families (AA7, CE8, CE12, GH2, GH28, GH35, GH43, GH51, GH53, GH54, GH78, GH105, GH127, GH139, and PL1) were upregulated under pectin treatment, and another eight genes of six families (AA7, GH31, GH43, GH127, GH2, and GH54) were downregulated (Table 2). Interestingly, the GH31 family only contains genes that were downregulated. The reason could be that the GH31 genes in Table 2 target other plant polysaccharides such as hemicelluloses and starches instead of pectins, as it is a large protein family with enzymes characterized with diverse substrates (48).

In addition, more CAZyme families might be involved in pectin degradation according to recent papers (33, 46), such as CE13, PL2, PL3, PL4, PL9, PL10, PL11, PL22, PL26, PL28, GH16, GH93, GH106, GH140, and GH141. However, these families were either not found in the TS63-9 genome according to dbCAN3 or were not differentially expressed under pectin treatment. The only exception is the GH16 family, which was mentioned to be involved in pectin degradation as galactan endo-β-1,3-galactosidases (EC 3.2.1.181) (33). There are 19 GH16 genes encoded in the TS63-9 genome (Table S3). While six of them were differentially expressed under pectin treatment, none were upregulated (Table S2). Like GH31, GH16 is also a large protein family with enzymes characterized with diverse substrates (49). Six biochemically characterized GH16 proteins are listed on the CAZy webpage (19) with the EC 3.2.1.181. Beta-galactan exists in both pectins (RG-I) in the side chain and arabinogalactan proteins (AGPs) in the backbone chain. However, reviewing the PubMed references listed on the CAZy webpage for these six proteins found that all of them have AGPs as the substrates. Therefore, we have excluded GH16 from our list of pectin-degrading CAZyme families.

Based on the presence of CAZyme families and the differential gene expression analysis, we propose a model to illustrate the roles of CAZyme families in pectin degradation in TS63-9 (Fig. 5). This model is consistent with our current knowledge of pectinases summarized in recent papers and agrees with dbCAN/CAZy annotation (19, 20, 33, 46, 47). For instance, the HG and RG-II backbones are mainly targeted by GH28 (polygalacturonases and rhamnogalacturonases), PL1 (pectate lyase), and AA7 (galacturonide oxidases), while the RG-I backbone is targeted by GH28, GH78 (rhamnohydrolases), and GH105 (rhamnohydrolases) for their degradation. The side chains of RG-I require GH35 (galactanases), GH53 (galactanases), GH51 (arabinofuranosidases), GH54 (arabinofuranosidases), and GH43 (endo-arabinanases), while the side chains of RG-II require GH2 (glucuronidases), GH28 (rhamnogalacturonases), GH78 (rhamnohydrolases), GH127 (arabinofuranosidase), GH139 (fucosidase), and GH43 (arabinofuranosidases) for their degradation. Finally, the two pectin acetylesterases (CE12 and CE13) are also included in the model.

Among all these families, the GH28 family is the most interesting, encoding polygalacturonases (PGs with EC 3.2.1.15, 3.2.1.67, and 3.2.1.171) and rhamnogalacturonases, as it has the most genes with the highest fold change under pectin treatment (Table 2). The 11 pectin-induced GH28 genes in TS63-9 encode diverse activities: six endo-PGs with EC 3.2.1.15 (ZTR_10728, ZTR_03993, ZTR_11349, ZTR_10118, ZTR_11088, and ZTR_08527), one exo-PG with EC 3.2.1.67 (ZTR_03139), one endo-α−1,2-galacturonidase with EC 3.2.1.171 (ZTR_08398), one endo-xylogalacturonan hydrolase with EC 3.2.1.- (ZTR_04324), one galacturan 1,4-α-galacturonidase (ZTR_00193), and one α-L-rhamnosidase (ZTR_09370), according to their best hits in the CAZy database (19). We also experimentally verified the functional role of the endo-α−1,4-galacturonidase (endo-PG) gene ZTR_10118 (3.2.1.15) in pectin degradation through a heterologous expression experiment (Fig. 4). This expansion of GH28 PGs, also observed in other phytopathogens such as *Sclerotinia sclerotiorum* and *Botryotinia fuckeliana* (50), underscores their crucial role in pectin decomposition.

TS63-9 is not only equipped with the 39 upregulated pectin-degrading CAZyme genes for extracellular breakdown of pectins but also 30 IPCGs (21). These upregulated IPCGs (Table S4) are responsible for the intracellular catabolism of pectin breakdown products, encoding key enzymes in the intracellular sugar-specific metabolic pathways (e.g., D-galactoses, D-mannoses, D-gluconates, D-galacturonic acids, and L-rhamnoses), the PCP, PPP, as well as the central carbon catabolic pathways (e.g., glycolysis, TCA, and glyoxylate cycles).

Interestingly, a computational promoter analysis found that the coordinated upregulation of 15 pectin-degrading CAZyme genes and 11 IPCGs might be controlled by a putative *cis*-regulatory motif in their promoter regions (Table 3). This conserved motif (**CCACCA**) may represent a putatively new transcriptional regulatory element coordinating fungal pectinase expression (51–54). Its presence across multiple enzyme classes (polygalacturonases, rhamnogalacturonases, pectate lyases, and enzymes of various intracellular pectin catabolic pathways) and fungal species raises the possibility of a conserved regulatory mechanism for pectin catabolism. The observed positional flexibility (51–871 bp) and sequence variations (Table 3) reflect evolutionary adaptations for differential gene regulation. If validated experimentally, it could potentially inform *T. verruculosus* strain engineering for industrial production of pectinolytic enzymes. However, we emphasize that the putative pectin-responsive motif requires future experimental confirmation (e.g., via promoter-reporter assays or electrophoretic mobility shift assays).

One limitation of this study is the absence of a carbon starvation control (e.g., no carbon source), as previous work has demonstrated that carbon starvation can induce the expression of plant cell wall-degrading enzymes, including pectinases, in filamentous fungi (55). Therefore, we cannot exclude the possibility that some of the pectinase genes identified in this study may be partially upregulated in response to carbon limitation rather than specifically induced by pectin.

### Conclusions

In this study, we sequenced the transcriptomes of *T. verruculosus* TS63-9 and identified 1,435 differentially expressed genes in response to pectin treatment. These DEGs include 671 upregulated and 764 downregulated genes. Functional annotation classified these upregulated genes primarily into categories of carbohydrate transport and metabolism, energy production and conversion, and amino acid transport and metabolism. Notably, pectin treatment specifically induced the upregulation of 39 pectin-degrading CAZyme genes and 30 IPCGs. Among them, 18 encoded secreted CAZymes of different GH, CE, and PL families, suggesting the formation of an elaborate extracellular pectinolytic network in strain TS63-9. The successful heterologous expression of an endo-PG gene (ZTR_10118) in yeast demonstrates the biotechnological potential of these enzymes. Collectively, our findings provide novel insights into the pectin degradation system in *T. verruculosus* TS63-9.

## Data Availability

The RNA-seq data of *T. verruculosus* TS63-9 are available at GenBank under the BioProject ID: PRJNA1214872, including the raw reads (SRX27451066–SRX27451071) and processed expression values (GEO: GSE331034) in the six samples. The genome assembly (GCA_001305275.1) of *T. verruculosus* TS63-9 and its gene annotation, published in 2016 (17), are available at GenBank under the BioProject ID: PRJNA291496.

## SUPPLEMENTAL MATERIAL

Figure S1 spectrum.04069-25-s0001.pdf Phylogeny of 18S rRNA sequences.

Figure S2 spectrum.04069-25-s0002.pdf Sequence logo of the CCACCA motif with two flanking bases up and downstream.

Legends spectrum.04069-25-s0003.docx Supplemental material legends.

Table S1 spectrum.04069-25-s0004.docx Expression of all 11,326 genes in 6 samples of *T. verruculosus* TS63-9.

Table S2 spectrum.04069-25-s0005.xlsx Differentially expressed genes in *T. verruculosus* TS63-9 following pectin treatment.

Table S3 spectrum.04069-25-s0006.xls dbCAN3 annotation (HMMER search against dbCAN HMMdb and dbCAN_sub HMMdb).

Table S4 spectrum.04069-25-s0007.xlsx Intracellular pectin catabolic genes (IPCGs) in *Aspergillus niger* and TS63-9.

Table S5 spectrum.04069-25-s0008.docx BLASTP alignment of KUL83159 and its best Swiss-Prot hit Q9Y833.

ASM does not own the copyrights to Supplemental Material that may be linked to, or accessed through, an article. The authors have granted ASM a non-exclusive, world-wide license to publish the Supplemental Material files. Please contact the corresponding author directly for reuse.
