# Supplementary material for: Transcriptome study of Talaromyces verruculosus TS63-9 for pectin-degrading CAZymes
Source: Microbiol Spectr. 2026 Jul 13;14(8):e04069-25. doi: 10.1128/spectrum.04069-25 (PMC13436326; doi:10.1128/spectrum.04069-25)
Supplement: Supplementary file 1 [file spectrum.04069-25-s0001.pdf]

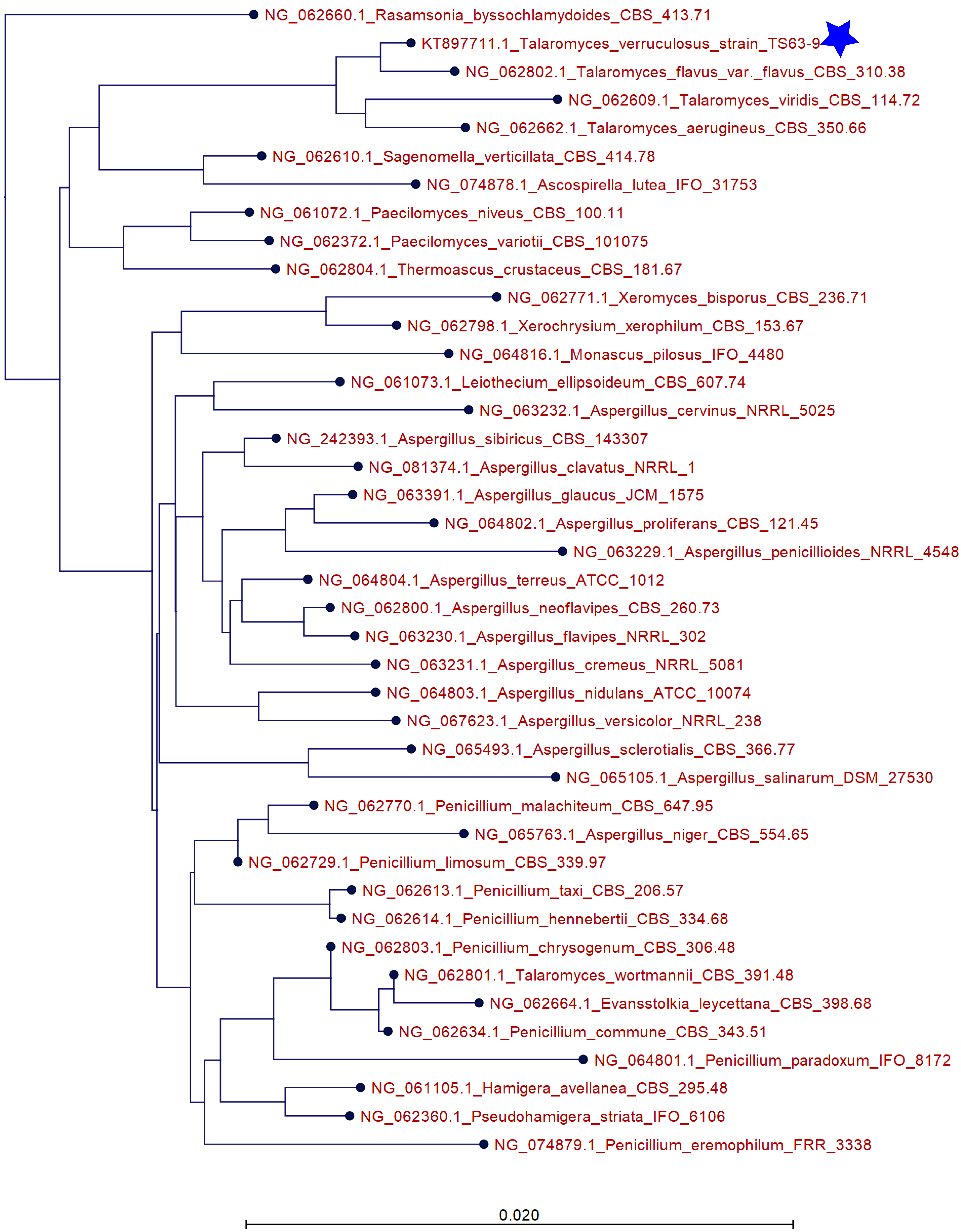

NG_062660.1_Rasamsonia_byssochlamydoides_CBS_413.71
KT897711.1_Talaromyces_verruculosus_strain_TS63-9
NG_062802.1_Talaromyces_flavus_var._flavus_CBS_310.38
NG_062609.1_Talaromyces_viridis_CBS_114.72
NG_062662.1_Talaromyces_aerugineus_CBS_350.66
NG_062610.1_Sagenomella_verticillata_CBS_414.78
NG_074878.1_Ascospirella_lutea_IFO_31753
NG_061072.1_Paecilomyces_niveus_CBS_100.11
NG_062372.1_Paecilomyces_variotii_CBS_101075
NG_062804.1_Thermoascus_crustaceus_CBS_181.67
NG_062771.1_Xeromyces_bisporus_CBS_236.71
NG_062798.1_Xerochrysium_xerophilum_CBS_153.67
NG_064816.1_Monascus_pilosus_IFO_4480
NG_061073.1_Leiothecium_ellipsoideum_CBS_607.74
NG_063232.1_Aspergillus_cervinus_NRRL_5025
NG_242393.1_Aspergillus_sibiricus_CBS_143307
NG_081374.1_Aspergillus_clavatus_NRRL_1
NG_063391.1_Aspergillus_glaucus_JCM_1575
NG_064802.1_Aspergillus_proliferans_CBS_121.45
NG_063229.1_Aspergillus_penicillioides_NRRL_4548
NG_064804.1_Aspergillus_terreus_ATCC_1012
NG_062800.1_Aspergillus_neoflavipes_CBS_260.73
NG_063230.1_Aspergillus_flavipes_NRRL_302
NG_063231.1_Aspergillus_cremeus_NRRL_5081
NG_064803.1_Aspergillus_nidulans_ATCC_10074
NG_067623.1_Aspergillus_versicolor_NRRL_238
NG_065493.1_Aspergillus_sclerotialis_CBS_366.77
NG_065105.1_Aspergillus_salinarum_DSM_27530
NG_062770.1_Penicillium_malachiteum_CBS_647.95
NG_065763.1_Aspergillus_niger_CBS_554.65
NG_062729.1_Penicillium_limosum_CBS_339.97
NG_062613.1_Penicillium_taxi_CBS_206.57
NG_062614.1_Penicillium_hennebertii_CBS_334.68
NG_062803.1_Penicillium_chrysogenum_CBS_306.48
NG_062801.1_Talaromyces_wortmannii_CBS_391.48
NG_062664.1_Evansstolkia_leycettana_CBS_398.68
NG_062634.1_Penicillium_commune_CBS_343.51
NG_064801.1_Penicillium_paradoxum_IFO_8172
NG_061105.1_Hamigera_avellanea_CBS_295.48
NG_062360.1_Pseudohamigera_striata_IFO_6106
NG_074879.1_Penicillium_eremophilum_FRR_3338
0.020
