# Supplementary material for: Transcriptome study of Talaromyces verruculosus TS63-9 for pectin-degrading CAZymes
Source: Microbiol Spectr. 2026 Jul 13;14(8):e04069-25. doi: 10.1128/spectrum.04069-25 (PMC13436326; doi:10.1128/spectrum.04069-25)
Supplement: Supplementary file 2 [file spectrum.04069-25-s0002.pdf]

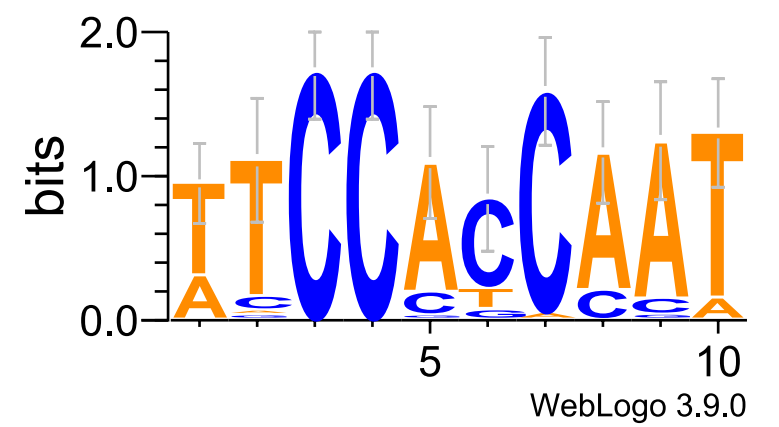
2.0
1.0
0.0
bits
5
10
WebLogo 3.9.0
