# Supplementary material for: Transcriptome study of Talaromyces verruculosus TS63-9 for pectin-degrading CAZymes
Source: Microbiol Spectr. 2026 Jul 13;14(8):e04069-25. doi: 10.1128/spectrum.04069-25 (PMC13436326; doi:10.1128/spectrum.04069-25)
Supplement: Supplementary file 3 [file spectrum.04069-25-s0003.docx]

Supplementary Materials

**Table S1**: Expression of all 11,326 genes in 6 samples of *T. verruculosus* TS63-9.

**Table S2**: Differentially expressed genes in *T. verruculosus* TS63-9 following pectin treatment.

**Table S3:** dbCAN3 annotation (HMMER search against dbCAN HMMdb and dbCAN_sub HMMdb).

**Table S4:** Intracellular pectin catabolic genes (IPCGs) in Aspergillus niger and TS63-9.

**Table S5**: BLASTP alignment of KUL83159 and its best Swiss-Prot hit Q9Y833.

**Figure S1**. Phylogeny of 18S rRNA sequences. *T. verruculosus* TS63-9 18S rRNA (KT897711.1) and their top hits in GenBank are used to build the phylogeny. All nodes have bootstrap values > 80%.

**Figure S2**. Sequence logo of the CCACCA motif with two flanking bases up and downstream. The logo was created using the motif sequences shown in Table 3 using WebLogo v3.9.0 (1).

1. Crooks GE, Hon G, Chandonia JM, Brenner SE. 2004. WebLogo: a sequence logo generator. Genome Res 14:1188–1190. https://doi.org/10.1101/gr.849004
