# Supplementary material for: Transcriptome study of Talaromyces verruculosus TS63-9 for pectin-degrading CAZymes
Source: Microbiol Spectr. 2026 Jul 13;14(8):e04069-25. doi: 10.1128/spectrum.04069-25 (PMC13436326; doi:10.1128/spectrum.04069-25)
Supplement: Supplementary file 5 [file spectrum.04069-25-s0008.docx]

```
Table 5: BLASTP alignment of KUL83159 and its best Swiss-Prot hit Q9Y833

RID: 01GYMZMS114
Job Title:KUL83159:hypothetical protein ZTR_10118 [Talaromyces...
Program: BLASTP
Query: hypothetical protein ZTR_10118 [Talaromyces verruculosus] ID: KUL83159.1(amino acid) Length: 375
Subject:RecName: Full=Polygalacturonase 2; Short=PG 2; AltName: Full=Pectinase 2; Flags: Precursor [Penicillium olsonii] ID: Q9Y833.1(amino acid) Length: 380

Clusters producing significant alignments:
                                                                  #          #          Scientific      Cluster                    Max    Total Query   E   Per.   Acc.
Cluster Rep.                                                      Mem.       Taxa       Name            Ancestor        Taxid      Score  Score cover Value Ident  Len        Accession
RecName: Full=Polygalacturonase 2; Short=PG 2; AltName:...        0          0          Penicillium ... NA              99116      532    532   98%   0.0   75.67  380        Q9Y833.1

Alignments:

>RecName: Full=Polygalacturonase 2; Short=PG 2; AltName: Full=Pectinase 2; Flags: Precursor [Penicillium olsonii]
Sequence ID: Q9Y833.1 Length: 380
>polygalacturonase 2 [Penicillium olsonii]
Sequence ID: CAB46909.1 Length: 380
Range 1: 7 to 380

Score:532 bits(1371), Expect:0.0,
Method:Compositional matrix adjust.,
Identities:283/374(76%), Positives:321/374(85%), Gaps:5/374(1%)

Query  7    LAGLAVFSSVAL-AAPAPA---VTAPATLENRAASCTFSGSNGAASASKSKTSCSTIVLS  62
            L  L +F ++A+ A P PA   VTA   LE RA SCTFSGS GA+SASKSKT+C+TIVLS
Sbjct  7    LLMLGLFGALAVHALPEPAKAQVTAAPKLEERATSCTFSGSAGASSASKSKTACATIVLS  66

Query  63   SVAVPSGTTLDLTGLNSGTKVIFEGTTTFGYKEWSGPLVSVSGTDITVEGASGAVLDGDG  122
            +VAVPSGTTLDLTGLN GT VIFEG TTFGYKEWSGPLVSVSGTDITV+GASGA L+GDG
Sbjct  67   AVAVPSGTTLDLTGLNDGTTVIFEGETTFGYKEWSGPLVSVSGTDITVKGASGATLNGDG  126

Query  123  ARWWDGKGSNGGKTKPKFFYAHNLKSSSINNIKILNSPVQVFSINGADTLTLDHITIDNS  182
            +RWWDGKGSNGGKTKPKFFYAH + SS+I++I I+NSPVQVFSINGA  LTL  IT+DN
Sbjct  127  SRWWDGKGSNGGKTKPKFFYAHKMFSSTISDIHIVNSPVQVFSINGATDLTLSGITVDNR  186

Query  183  DGDS-AGGHNTDAFDVGSSNGVTISNANVQNQDDCLAVNSGTNIIFTGGTCSGGHGLSIG  241
            DGD+  GGHNTDAFDVGSS G+TI+ A V NQDDCLAVNSGT+I FTGG CSGGHGLSIG
Sbjct  187  DGDTDEGGHNTDAFDVGSSTGITITGATVYNQDDCLAVNSGTDITFTGGLCSGGHGLSIG  246

Query  242  SVGGRSDNTVDTVHIENSNIKNSQNGVRIKTVYDATGSVSGVTYKNITLSGITKYGIVIE  301
            SVGGRS+N V  V IENS I++S NGVRIKTVY+ATGSV  VTYK+ITLSGITKYGIVIE
Sbjct  247  SVGGRSNNDVANVIIENSQIQDSTNGVRIKTVYEATGSVKNVTYKDITLSGITKYGIVIE  306

Query  302  QDYENGSPTGTPTTGVPITGLTIDGVTGSVTSSATDVYILCGKGSCSNWTWEGVSITGGK  361
            QDYENGSPTGTPT GVPIT LT+DGV G+V SS T+VYILC +G+CS+WTW+GVS++GGK
Sbjct  307  QDYENGSPTGTPTDGVPITDLTLDGVKGTVASSGTNVYILCAEGACSDWTWDGVSVSGGK  366

Query  362  KSTGCKNIPSGASC  375
             S+ C+N+PS ASC
Sbjct  367  TSSACENVPSSASC  380
```
